# Molecular evidence for natural hybridization between *Rumex crispus* and *R. obtusifolius* (Polygonaceae) in Korea

**DOI:** 10.1038/s41598-022-09292-9

**Published:** 2022-03-31

**Authors:** Gauri Shankar Bhandari, Chong-Wook Park

**Affiliations:** School of Biological Sciences, Seoul National University, Seoul, 08826 Korea

**Keywords:** Evolution, Plant sciences

## Abstract

Interspecific hybridization has been suggested to occur frequently in *Rumex* (Polygonaceae). Several hypothesized combinations of parental species of hybrids based on their intermediate morphology have been suggested in the genus, but few of them have been phylogenetically tested. We analyzed nuclear and chloroplast DNA sequence data of a putative natural hybrid between *Rumex crispus* and *Rumex obtusifolius* from Korea to confirm its hybrid status and to determine the maternal parent. Analysis of the nuclear DNA *pgiC* region revealed that *R. crispus* and *R. obtusifolius* have contributed to the nuclear genome of the putative hybrids. The haplotype distribution pattern inferred from the combined sequence data set of five chloroplast DNA regions (*matK, rbcL-accD* IGS*, trnK-rps16* IGS*, ycf6-psbM* IGS and *psbA-trnH* IGS) indicated bidirectional hybridization events between *R. crispus* and *R. obtusifolius*. This paper provides the first molecular evidence for interspecific hybridization between *R. crispus* and *R. obtusifolius*. In addition, our findings strongly suggested that Korean populations of *Rumex japonicus* have a hybrid origin, and *R. crispus* may represent one of the parental taxa.

## Introduction

The widespread occurrence of interspecific hybridization within *Rumex* L. (Polygonaceae) suggests that it may play a significant role in speciation and evolution in the genus^1–4^. *Rumex* comprises approximately 200 species commonly known as docks and sorrels in colloquial English^5–7^. It has a nearly worldwide distribution, but most species occur in temperate regions of both hemispheres, and some have become naturalized beyond their native range^3,6^. The genus has been classified into four subgenera, *Rumex*, *Acetosa* Raf., *Acetosella* Raf., and *Platypodium* (Willk.) Rech.f. Although interspecific hybrids have been reported frequently in subgenus *Rumex*^3,8^, hybridization has not been recorded between species in different subgenera^3^. Hybrids are often morphologically intermediate between the putative parents and generally exhibit partial or almost complete sterility. Spontaneous hybrids between species of *Rumex* are usually less fertile and ecologically successful than the parental species^6^.

Among the species of *Rumex, R. crispus* L. is the most widespread and ecologically most successful. It is native to Europe and southwestern Asia, but occurs almost worldwide as a fully naturalized and sometimes invasive alien^6,8^. *Rumex obtusifolius* L. usually coexists with *R. crispus* and likewise has a global distribution. *Rumex obtusifolius* is also native to Europe and southwestern Asia, but it is also naturalized in many other parts of the world, including southern Africa, South and North America, and south and northeast Asia and Australia^6,8,9^^.^ Naturally occurring hybrids between *R. crispus* and *R. obtusifolius* are the most common in the genus, at least in Europe, and occur frequently wherever the parental species grow together, even in their naturalized ranges, including Australia and North America^1,2,6,8^. *Rumex japonicus* Houtt., which is distributed across northeast Asia including China, Korea, Japan and Far East Russia, frequently cooccurs with either *R. crispus* or *R. obtusifolius* separately or occasionally together.

Based on field observations and measurements, previous studies have indicated the occurrence of bidirectional introgression between *R. crispus* and *R. obtusifolius*^1,2^. In those studies, the hybrids exhibited characteristics of both parents in widely varying proportions ranging from individuals morphologically more similar to *R. crispus* to individuals that are nearly indistinguishable from *R. obtusifolius*. Backcrossing occurs with both parents, but is likely more common with *R. crispus* than with *R. obtusifolius*^1^. Backcrosses with *R. crispus* have been produced experimentally but have not been confirmed with *R. obtusifolius*^3^. In addition, hybrids are less fertile than the parents^2^.

Both *R. crispus* and *R. obtusifolius* are considered invasive alien species in Korea^7,10,11^. Such invasive plants can have significant negative impacts on the abundance and diversity of native species^12,13^. Plant invasions may also occur through hybridization. Invasive species can reduce genetic diversity and cause the extirpation of locally adapted populations of native species through hybridization, which is of particular concern for rare and threatened native species. Further, introgression with native relatives may give rise to more aggressive hybrid types, and the spread of aggressive hybrid taxa can reduce the fitness of, or replace, native species^14^.

Hybrid assessment in plants has been traditionally determined by using several criteria based on morphology, distribution, and crossing experiments^15–17^. More recently, various molecular methods have been used in determining hybridization events in higher plants^18–21^. Chloroplast DNA (cpDNA) is usually maternally inherited in angiosperms, so that it can be used to identify the maternal parent of putative hybrids^22–24^. The application of biparentally inherited, single- or low-copy nuclear DNA (nDNA) regions in combination with appropriate cpDNA regions has become an efficient way to validate hybridization events in plants^25–27^^.^ By utilizing such methods, hybrids have been proposed and validated in many genera, including *Melastoma* L., *Eriobotrya* Lindl., *Ilex* L., and *Microsorum* Link^28–31^. However, only a few attempts have been made to characterize hybrids in *Rumex* using molecular methods^4^.

**Figure 1:**
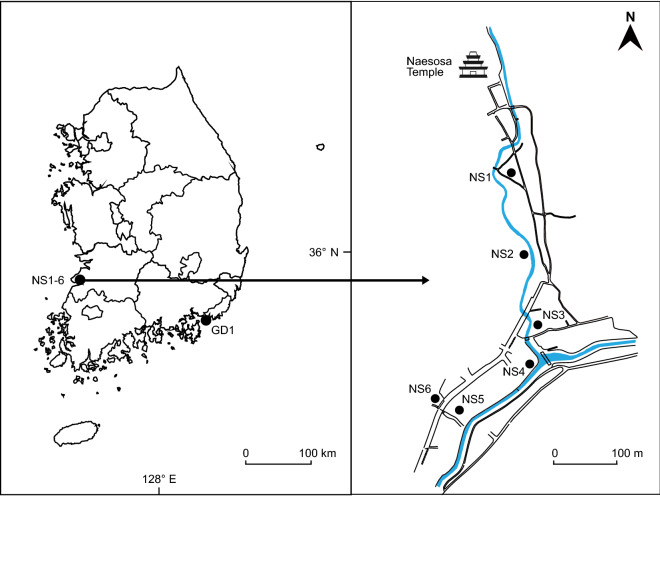
Geographic location of seven mixed populations of species of *Rumex* and putative hybrids in Korea sampled for this study. Six populations (NS1–NS6) were in the Naesosa area of Jeon-buk Province. One population (GD1) was on Gadeok Island, Busan. Naesosa and Gadeok Island are separated by over 200 km.

In Korea, 12 species of *Rumex* comprising three subgenera, *Rumex* (10 species), *Acetosa* (one species) and *Acetosella* (one species), have been reported^7^. In our investigation of natural hybridization among the species of subgenus *Rumex* across their entire range in South Korea, we found several plants with intermediate morphology in mixed populations of *R. crispus* and *R. obtusifolius* in seven localities (Fig. 1). Those observations prompted us to investigate further by employing low copy nDNA *pgiC* gene and five cpDNA regions (*matK, rbcL-accD* IGS*, trnK-rps16* IGS*, ycf6-psbM* IGS and *psbA-trnH* IGS) to assess their hybrid status and potential introgression.

## Results

### Chloroplast DNA

The sequence characteristics of the examined cpDNA regions, *matK, rbcL-accD* IGS*, trnK-rps16* IGS*, ycf6-psbM* IGS and *psbA-trnH* IGS*,* are summarized in Table 1. The combined cpDNA data set was 4007 base pairs (bp) in length after alignment (Table 1). There were 259 (6.5%) variable characters, 108 (2.7%) of which were parsimony informative (Table 1).

**Table 1:** Statistics for cpDNA and nDNA sequence data sets used in this study. Numbers in parentheses represent comparisons of ingroup taxa only.

	cpDNA						nDNA
	*matK*	*rbcL-accD* IGS	*trnK*-*rps16* IGS	*ycf*6-*psbM* IGS	*psbA*-*trnH* IGS	Combined cpDNA	*pgiC*
Sequence length (bp)	961–967	463–475	765–895	918–1094	328–369	3623–3646	962–1249
Aligned length (bp)	967	508	967	1147	418	4007	1387
No. of variable sites	37 (2)	28 (0)	79 (3)	79 (3)	36 (6)	259 (14)	284 (107)
No. of parsimony informative characters	23 (2)	6 (0)	28 (3)	34 (3)	17 (6)	108 (14)	132 (86)
GC ratio (%)	33.6–33.9	31.9–32.5	26.4–27.4	30.8–32.5	28.7–33.2	30.5–31.6	34.9–37.7
Optimal model of sequence evolution	GTR + I	HKY	GTR + G	GTR	F81 + I	GTR + G	HKY + I

Based on the combined data set, six cpDNA haplotypes were identified from 70 accessions of *R. crispus*, *R. obtusifolius*, *R. japonicus* and the putative hybrids (Table 2, Supplementary Table S1). Four haplotypes (C1, C3–C5) were recovered from 17 accessions of *R. crispus* sampled for this study. Among those haplotypes, haplotypes C3–C5 were also detected in accessions from pure, non-mixed populations of *R. crispus*, but haplotype C1 was restricted to those from mixed populations in the Naesosa area (Table 2, Supplementary Table S1). In *R. crispus*, haplotype C5 was detected in one accession and haplotypes C3 and C4 were detected in three and four accessions, respectively. In contrast to *R. crispus*, a single haplotype (C2) was detected in all accessions of *R. obtusifolius*. A total of five haplotypes (C1–C5) were recovered from the putative hybrids. In *R. japonicus*, two haplotypes (C1 and C6) were detected (Table 2, Supplementary Table S1).

**Table 2:** Variable nucleotide sites in five cpDNA regions of *Rumex crispus*, *R. obtusifolius*, *R. japonicus* and putative hybrids.

Haplotype	Taxon	Variable nucleotide sites in alignment																																								
		*matK*		*rbcL-accD* IGS						*trnK-rps16* IGS			*ycf6-psbM* IGS														*psbA-trnH* IGS															
		0 5 4 4	0 6 3 2	1 4 1 1	2 4 1 1	3 4 1 1	4 4 1 1	5 4 1 1	1 9 2 1	6 4 6 1	1 9 0 2	6 1 2 2	1 9 0 3	2 9 0 3	3 9 0 3	4 9 0 3	5 9 0 3	6 9 0 3	3 8 1 3	8 1 3 3	6 2 3 3	6 5 5 3	7 5 5 3	8 5 5 3	9 5 5 3	0 6 5 3	6 6 7 3	8 6 7 3	9 6 7 3	0 7 7 3	1 7 7 3	3 7 7 2	3 7 7 3	4 7 7 3	5 7 7 3	6 7 7 3	3 1 8 3	8 1 8 3	2 6 8 3	4 8 8 3	5 0 9 3	7 0 9 3
C1	*R. crispus, R. japonicus*, *R. crispus* x *R. obtusifolius*	T	G	–	–	–	–	–	–	G	T	T	–	–	–	–	–	–	T	A	A	C	A	T	T	A	A	–	–	–	–	–	–	–	–	–	A	T	G	T	T	A
C2	*R. obtusifolius*, *R. crispus* x *R. obtusifolius*	T	T	T	T	G	C	G	–	A	T	C	A	T	A	T	G	T	C	G	G	C	A	T	T	A	A	–	–	–	–	–	–	–	–	–	G	–	G	G	T	A
C3	*R. crispus*, *R. crispus* x *R. obtusifolius*	T	T	–	–	–	–	–	–	A	T	C	A	T	A	T	G	T	C	G	G	C	A	T	T	A	C	–	–	–	–	–	–	–	–	–	G	–	G	G	T	A
C4	*R. crispus*, *R. crispus* x *R. obtusifolius*	T	T	–	–	–	–	–	–	A	T	C	A	T	A	T	G	T	C	G	G	C	A	T	T	A	A	–	–	–	–	–	–	–	–	–	G	–	G	G	T	A
C5	*R. crispus, R. crispus* x *R. obtusifolius*	A	G	–	–	–	–	–	T	A	G	T	A	T	A	T	G	T	C	G	G	–	–	–	–	–	A	T	G	T	T	A	T	G	A	G	G	–	A	G	G	G
C6	*R. japonicus*	A	G	–	–	–	–	–	T	A	G	T	A	T	A	T	G	T	C	G	G	C	A	T	T	A	A	T	G	T	T	A	T	G	A	G	G	–	A	G	G	G

The distribution of cpDNA haplotypes in six populations in the Naesosa (NS1–NS6) area and one population on Gadeok Island (GD1) was examined (Supplementary Table S1). In populations NS1 and NS6, *R. crispus* contained haplotype C1, whereas the putative hybrids exhibited either haplotype C2 (three accessions) or C5 (five accessions). *Rumex japonicus* in population NS1 contained haplotype C1. In population NS2, *R. crispus* possessed either haplotype C1 (three accessions) or C4 (one accession), whereas all seven accessions of the putative hybrids exhibited haplotype C2. *Rumex japonicus* possessed either haplotype C1 (one accession) or C6 (two accessions). Two accessions of *R. crispus* sampled from population NS3 possessed haplotype C1. Haplotype C2 occurred in two putative hybrid individuals sampled from the same population. *Rumex japonicus* in this population exhibited haplotype C6 (Supplementary Table S1).

In population NS4, four haplotypes (C1, C3, C4 and C5) were detected in six accessions of *R. crispus* (Supplementary Table S1). Those same haplotypes were shared by nine accessions of the putative hybrids sampled from the same population. Three other accessions of the putative hybrids had haplotype C2. In particular, haplotype C3 was not found in any other populations in the Naesosa area, but was detected in accessions of *R. crispus* and the putative hybrids from the distantly located population on Gadeok Island (GD1). In population NS5, *R. crispus* possessed haplotype C1, whereas the putative hybrids possessed haplotypes C1 (three accessions), C2 (one accession) or C5 (two accessions). In population NS6, *R. crispus* possessed haplotype C1, whereas the putative hybrids possessed haplotypes C2 (two accessions) or C5 (two accessions). In population GD1 of Gadeok Island, accessions of both *R. crispus* and the putative hybrids shared haplotype C3; no putative hybrids shared a cpDNA haplotype with *R. obtusifolius* (Supplementary Table S1).

### Nuclear DNA *pgiC*

A total of 85 sequences of the nDNA *pgiC* region was recovered from 57 accessions representing *R. crispus*, *R. obtusifolius*, *R. japonicus* and the putative hybrids (Fig. 3). In addition, we examined the *pgiC* sequences of nine accessions of *R. crispus* and six of *R. obtusifolius* obtained from pure, non-mixed populations of each species (Supplementary Table S1). The length of *pgiC* was 978 bp in *R. crispus*, 996 bp in *R. obtusifolius*, 968–1249 bp in *R. japonicus*, and 978–996 bp in the putative hybrids. The length of the *pgiC* data set after alignment was 1387 bp (Table 1). There were 284 (20.5%) variable characters, 132 (9.5%) of which were parsimony informative (Table 1).

All accessions of *R. crispus* and *R. obtusifolius* and eight accessions of the putative hybrids had a single *pgiC* sequence type, but 19 accessions of the putative hybrids had two sequence types. Accessions of *R. japonicus* had two or three sequence types (Fig. 3). Five *pgiC* haplotypes (N1–N5) were identified from 57 accessions of *R. crispus*, *R. obtusifolius*, *R. japonicus,* and the putative hybrids (Fig. 3, Supplementary Table S1). All accessions of *R. crispus* exhibited haplotype N2, whereas those of *R. obtusifolius* possessed haplotype N3. Four haplotypes (N1, N2, N4 and N5) were detected in *R. japonicus*. Accessions of the putative hybrids possessed either haplotype N2 or N3 only, or both N2 and N3 *pgiC* haplotypes (Fig. 3).

### Phylogenetic analyses

**Figure 2:**
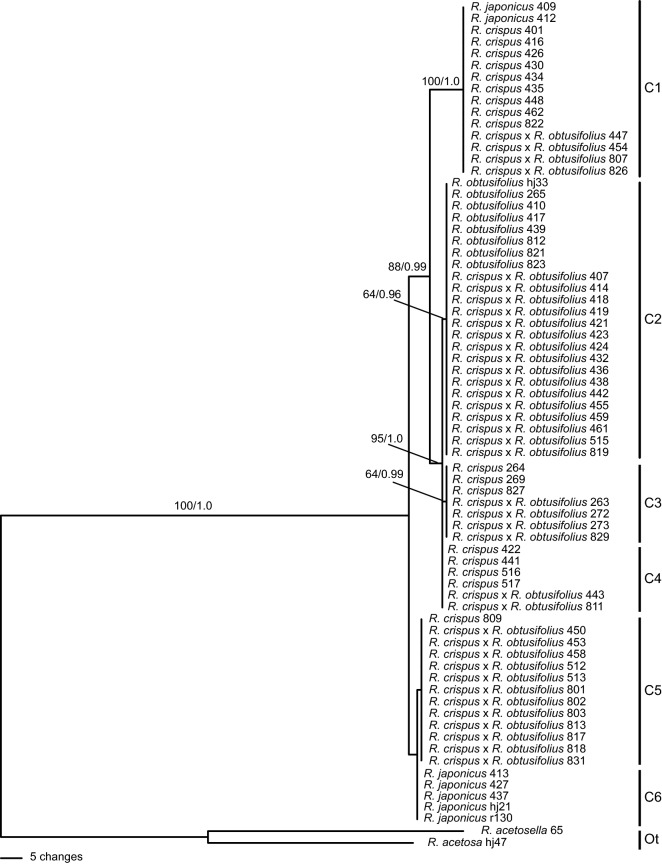
Most parsimonious tree from the maximum parsimony analysis of the combined cpDNA sequence data set for *Rumex crispus*, *R. obtusifolius*, *R. japonicus* and putative hybrids. Numbers above branches indicate MP bootstrap values (BS ≥ 50) and Bayesian posterior probabilities (PP ≥ 0.7). Accession numbers correspond to those in Supplementary Table S1. C1–C6 = cpDNA haplotypes; Ot = Outgroup.

The maximum parsimony (MP) analysis of the combined cpDNA data set resulted in a single most parsimonious tree with 278 steps (CI = 0.989, RI = 0.994) (Fig. 2). The majority-rule consensus tree obtained from Bayesian inference (BI) analysis of the same data set was basically identical to the MP tree in topology and groupings (Supplementary Fig. S1). In the cpDNA tree (Fig. 2), none of the species included in this study were resolved as monophyletic. Accessions of *R. obtusifolius* formed a weakly supported clade (BS = 64, PP = 0.96) with 16 accessions of the putative hybrids; these accessions shared haplotype C2. Also, accessions of *R. crispus* were not resolved as monophyletic; they formed widely separated clades either with accessions of the putative hybrids (clades with haplotype C3 or C5) or with those of the putative hybrids and *R. japonicus* (clade with haplotype C1). Four accessions of *R. crispus* and two accessions of the putative hybrids with haplotype C4 remained unresolved within the clade comprising accessions with haplotype C2 or C3. The accessions of the putative hybrids grouped with either *R. crispus* or *R. obtusifolius,* or with a clade including *R. crispus* and *R. japonicus* (Fig. 2). The results strongly suggested that hybridization has occurred in the populations sampled.

**Figure 3:**
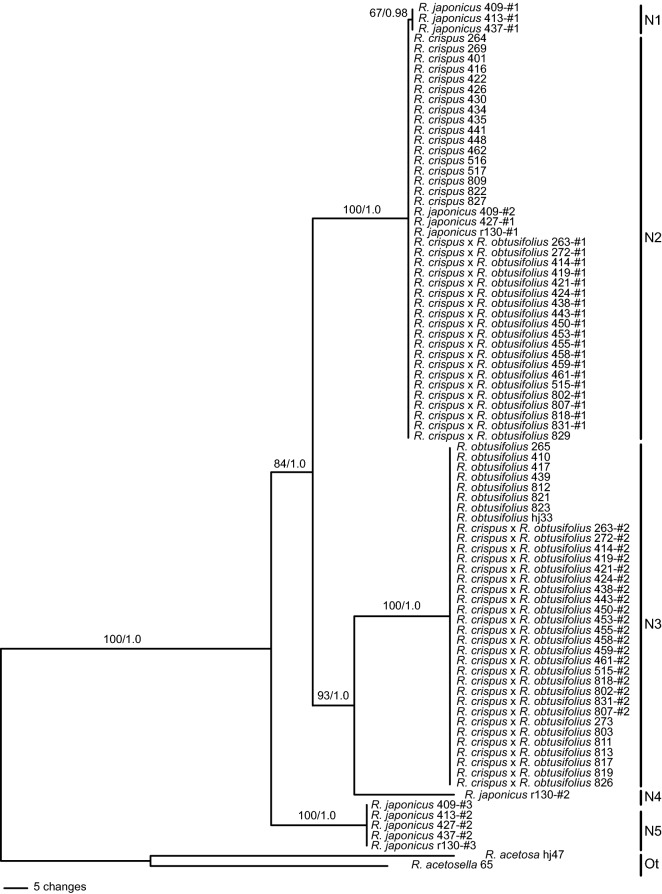
Most parsimonious tree from the maximum parsimony analysis of the nDNA *pgiC* sequence data set for *Rumex crispus*, *R. obtusifolius*, *R. japonicus* and putative hybrids. Numbers above branches indicate MP bootstrap values (BS ≥ 50) and Bayesian posterior probabilities (PP ≥ 0.7). Accession numbers correspond to those in Supplementary Table S1. Numbers following accession numbers are clone numbers. N1–N5 = nDNA *pgiC* haplotypes; Ot = Outgroup.

The MP analysis of the nDNA *pgiC* data set resulted in a single most parsimonious tree with 345 steps (CI = 0.939, RI = 0.990) (Fig. 3). The majority-rule consensus tree obtained from BI analysis of the same data set was identical to the MP tree in topology and groupings (Supplementary Fig. S2). In the nDNA tree (Fig. 3), four strongly supported clades (BS ≥ 93, PP = 1.0) were resolved; a clade consisting of haplotypes N1 from *R. japonicus* (three cloned accessions) and N2 from *R. crispus,* the putative hybrids (20 accessions; 19 cloned) and *R. japonicus* (three cloned accessions), (2) a clade representing haplotype N3 from *R. obtusifolius* and the putative hybrids (26 accessions; 19 cloned), (3) clade representing haplotype N4 from *R. japonicus* (one cloned accession), and (4) clade representing haplotype N5 from *R. japonicus* (five cloned accessions). Especially noteworthy is that all 19 cloned accessions of the putative hybrids recovered two haplotypes, N2 and N3, which were present in all accessions of *R. crispus* and *R. obtusifolius*, respectively. Eight directly sequenced accessions of the putative hybrids recovered either haplotype N2 or N3 (Fig. 3).

**Figure 4:**
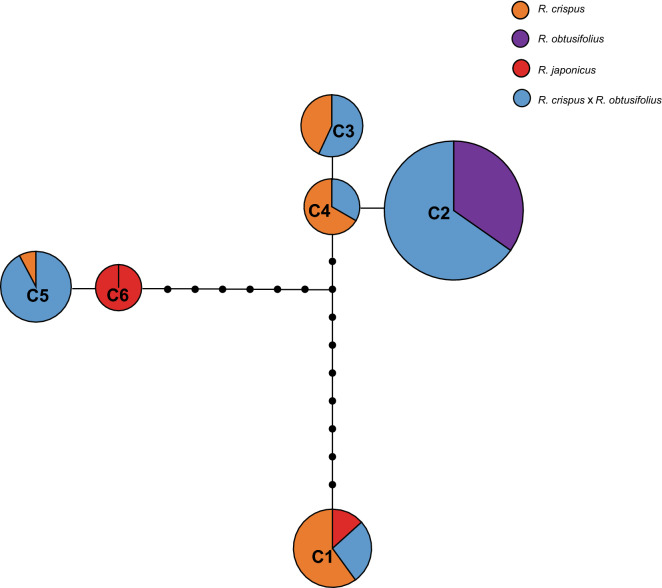
Statistical parsimony network of cpDNA sequences from *Rumex crispus*, *R. obtusifolius*, *R. japonicus* and putative hybrids. Each circle represents a haplotype; circle size is approximately proportional to haplotype frequency. Lines represent single mutation steps; small black circles represent inferred haplotypes but not present in samples.

The statistical parsimony analysis of the combined cpDNA sequences as implemented in TCS resulted in a network of six haplotypes (Fig. 4). Those haplotypes were separated by one to 16 mutations. Fifteen haplotypes inferred by TCS were not found in the analyzed individuals and occurred as missing haplotypes in the network. Four haplotypes (C1, C3, C4, and C5) belonged to *R. crispus*; all those haplotypes were shared with hybrid accessions. The haplotypes in *R. crispus* were separated by one (C3 and C4) to 16 (C1 and C5) mutation steps. Only one haplotype (C2) was found in *R. obtusifolius,* and it was shared with hybrid accessions. Haplotype C6 was found only in *R. japonicus*.

## Discussion

In the present study, we detected putative hybrid individuals with intermediate morphology from mixed populations of *R. crispus* and *R. obtusifolius* in seven localities in Korea (Fig. 1). The cloned nDNA *pgiC* sequences of the 19 accessions of the putative hybrids sampled from those populations revealed two types of sequences in each accession, haplotypes N2 and N3. Haplotypes N2 and N3 were exhibited by *R. crispus* and *R. obtusifolius*, respectively. Haplotype N2 differed from haplotype N3 by 54 bp substitutions, and there were no shared *pgiC* haplotypes between the two species (Fig. 3, Supplementary Table S2). The intermediate morphology and co-occurrence of two divergent *pgiC* haplotypes corresponding to *R. crispus* and *R. obtusifolius* indicated that those individuals were F1 hybrids between them. The putative hybrid accessions shared cpDNA haplotypes with either *R. crispus* or *R. obtusifolius,* suggesting that both species served as the maternal parent.

In comparison, eight directly sequenced accessions of the putative hybrids with intermediate morphology recovered either nDNA *pgiC* haplotype N2 (one accession) or N3 (seven accessions) (Fig. 3). Since these putative hybrid individuals occur only in mixed populations of the presumed parental species, and most of their flowers failed to set fruit, became dry and fell before any appreciable enlargement of the valves, it is highly likely that they represent backcross generation of F1 hybrids. Repeated backcrossing to either one of parental species may lead to elimination of one of the two copies initially present in the nuclear genome of F1 hybrids^32^. In particular, six putative hybrid accessions (273, 803, 811, 813, 817, 826) shared nDNA *pgiC* haplotype N3 with *R. obtusifolius*, but shared cpDNA haplotypes (C1, C3–C5) with *C. crispus* (Figs. 2, 3). The findings strongly suggested that backcrossing or introgression has occurred in these populations to at least some extent.

Maternal inheritance of chloroplasts, which is typical in angiosperms, has been experimentally demonstrated in the Polygonaceae^18^. If maternal chloroplast inheritance is also assumed to occur in *Rumex*, then the pattern of haplotype distribution revealed in our study suggests bidirectional hybridization events between *R. crispus* and *R. obtusifolius*. In population NS4, for example, nine accessions of the putative hybrids comprised four cpDNA haplotypes (C1, C3–C5), all of which were detected in *R. crispus* accessions from the same population. On the other hand, the other three accessions of the putative hybrids contained haplotype C2 detected in *R. obtusifolius* accessions also from the same population (Supplementary Table S1). The results strongly indicated that hybridization between the two species was bidirectional, and both *R. crispus* and *R. obtusifolius* served as either paternal or maternal parent. Bidirectional hybridization between *R. crispus* and *R. obtusifolius* has been suggested in previous studies^1,2^. Bidirectional hybridization in plants is relatively common and has been reported in several other plant taxa^18,33–35^.

The cpDNA haplotype C3 detected in population NS4 was not recovered from any other populations in the Naesosa area, but was detected in accessions of *R. crispus* and putative hybrids from the distantly located Gadeok Island population (GD1); populations NS4 and GD1 are separated by over 200 km. It is possible that we were unable to detect this haplotype in other populations in the Naesosa area due to the relatively small sample size. Conversely, the results may suggest the occurrence of long-distance seed dispersal. In *Rumex*, achenes are dispersed by wind or occasionally through excreta of mammals and birds^36^. Birds occasionally eat seeds of *R. crispus* when other quality food is not available, and seedlings have been raised from the excreta of various birds^36,37^.

The detection of four cpDNA haplotypes (C1, C3–C5) in only six accessions of *R. crispus* in population NS4 indicated that *R. crispus* is highly polymorphic for cpDNA haplotypes (Supplementary Tabel S1). The haplotype network revealed that haplotypes C1 and C5 possessed by the accessions *of R. crispus* in population NS4 were markedly different from each other and separated by 16 mutation steps (Fig. 4); this strongly suggested that haplotypes C1 and C5 probably diverged long ago. In contrast, all accessions of *R. crispus* from all populations had identical nuclear haplotypes. Since *R. crispus* invaded Korea relatively recently, the co-occurrence of highly divergent cpDNA haplotypes in populations of *R. crispus* may suggest repeated invasions from multiple sources with different genetic backgrounds^10,38^.

Although cpDNA haplotype C1 and nDNA *pgiC* haplotype N1 were shared by *R. japonicus, R. crispus* and some putative hybrid accessions, it is unlikely that *R. japonicus* participated in the formation of those hybrid individuals, as one of the two cpDNA haplotypes and three of the four nDNA *pgiC* haplotypes exhibited by *R. japonicus* were unique and not detected in any putative hybrid accessions (Figs. 2, 3, 4). In contrast, all the cpDNA and nDNA *pgiC* haplotypes of *R. crispus* were detected in the putative hybrids (Figs. 2, 3, 4). The results strongly suggested that *R. crispus*, rather than *R. japonicus*, served as one of the parental species of the putative hybrids.

The cloning of five accessions of *R. japonicus* recovered multiple *pgiC* sequence types; two accessions (r130, 409) had three sequence types (haplotypes N2, N5 and N1 or N4), and three (413, 427, 437) had two sequence types (haplotypes N1 or N2 and N5) (Fig. 3). Among the multiple *pgiC* haplotypes recovered from *R. japonicus*, haplotype N2 was shared by *R. crispus* (Fig. 3). *Rumex japonicus* possessed two cpDNA haplotypes, one of which was shared with *R. crispus*. Our findings suggested that Korean populations of *R. japonicus* have a hybrid origin and that *R. crispus* may represent one of the parental taxa. However, the hybrid status and parentage of the Korean populations of *R. japonicus* were beyond the scope of this study. Further studies are needed to validate the hybrid origin and parental taxa involved.

Putative hybrid individuals in all populations bore a few fruit containing potentially viable seeds. Molecular data supported the F1 hybrid or backcross nature of these individuals. The fertility of F1 hybrids and backcrosses between *R. crispus* and *R. obtusifolius* have been reported previously^1,2,39^. As such, putative hybrids and backcrosses may lead to the formation of hybrid swarms. Hybrid individuals between *R. crispus* and *R. obtusifolius* reported from Europe, Australia and North America have been referred to as *R.* x *pratensis* Mert. & W. D. J. Koch^1–3,8,40^.

Analyses of chloroplast and nuclear DNA sequence data in the present study provided compelling evidence for the occurrence of natural bidirectional hybridization between *R. crispus* and *R. obtusifolius*. The hybrids arise frequently in mixed populations of the two parental species in Korea. Confirmation of interspecific hybridization between recently introduced alien species such as *R. crisp*us and *R. obtusifolius* would be significant because of the possible ecological consequences of hybrids and hybrid swarms. The hybrid populations we sampled were located in disturbed sites including roadsides, abandoned fields, forest clearings, and riparian zones. Thus, it appears that habitat disturbance may favor the formation of interspecific hybrids in *Rumex*. Frequent hybridization between species of *Rumex* in disturbed habitats has been reported previously^1,2,4^. The possible existence of F2 progeny and backcross generations in *Rumex* have been reported in previous studies^2,4^. The fertility of backcross generations usually increases as compared to that of the F1 generation in *Rumex* and in several other angiosperms, including *Trifolium* L. and *Helianthus* L.^2,41,42^ The increased fertility of backcross generations in hybrid swarms may result in the loss of genetic integrity of the parental species. In addition, it is possible that F1 hybrids and backcross plants are more invasive than their parental species due to hybrid vigor. Consequently, those plants may negatively impact native biodiversity, especially native plants of grasslands and forest margins, by occupying similar habitats and competing for resources. We recommend further study to understand the ecological consequences of the hybrids and to clarify whether the genetic integrity of the parental species is maintained. In addition, further studies are needed to validate the hybrid origin of the Korean populations of *R. japonicus*.

## Materials and methods

### Taxon sampling

We sampled 17 individuals of *R. crispus*, eight individuals of *R. obtusifolius*, seven individuals of *R. japonicus* and 38 individuals of putative hybrids from six populations (NS1–NS6) in the Naesosa region, Jeon-buk Province, and one population (GD1) on Gadeok Island of Busan, Korea (Fig. 1, Supplementary Table S1). We also examined nine accessions of *R. crispus* and six of *R. obtusifolius* obtained from pure, non-mixed populations of each species to validate species-specific haplotypes of those species (Supplementary Table S1). Because it occurs in some mixed populations of *R. crispus* and *R. obtusifolius*, we included *R. japonicus* in our study to evaluate its potential contribution to the nuclear and/or chloroplast genome of the putative hybrids*.* The individuals were collected in July 2020 and June-July 2021. Naesosa (35° 37′ N 126° 35′ E) refers to the site of an ancient Buddhist temple in Byeonsanbando National Park on the west coast of South Korea (Fig. 1). It is located at the base of a mountain at an altitude of 30 m above sea level. The study sites in Naesosa were disturbed areas, including roadsides, abandoned agricultural fields, forest clearings and riparian zones. Gadeok (35° 02′ N 128° 49′ E) is an island in Busan, South Korea (Fig. 1). The sampled population on the island was on a roadside at the base of a mountain, at an altitude of about 40 m above sea level. Permission for collecting plant samples from the Naesosa area was obtained from Byeonsanbando National Park, Korea. The collection locations of the other plant samples were neither in protected areas nor on private land, and thus no permission was required for collections from these locations. *Rumex acetosa* L. (subgenus *Acetosa*) and *R. acetosella* L. (subgenus *Acetosella*) were selected as outgroups.

Initial identifications of the individuals collected from the above populations were carried out by the authors based on differences in the major morphological characters of *Rumex*. *Rumex crispus*, *R. obtusifolius* and *R. japonicus* are distinguished mainly on the basis of differences in the shape of the leaves and the valves of their fruit. The basal and lower cauline leaves of *R. crispus* are lanceolate or oblong-lanceolate with strongly crisped margins. The valves are broadly ovate with entire margins. In contrast, the basal and lower cauline leaves of *R. obtusifolius* are usually ovate to elliptic or ovate-oblong with entire margins, and the valves are triangular-ovoid with spinose margins. *Rumex japonicus* has lanceolate to oblanceolate leaves somewhat similar to those of *R. crispus*, but it can be distinguished from the other two species by its reniform or broadly pentagonal valves with irregularly and shallowly toothed margins. Hybrid individuals of *R. crispus* and *R. obtusifolius* are usually intermediate in leaf shape between the two parental species. Most flowers of hybrid individuals failed to set fruit; they became dry and fell before any appreciable enlargement of the valves. A few flowers appeared to be fertile and set fruit, with the valves enlarging to varying degrees. All voucher specimens were deposited in Seoul National University Herbarium (SNU) with Herbarium IDs: 119,355–119,426. All experiments and methods were performed in accordance with relevant regulations and guidelines.

### DNA extraction, amplification, and sequencing

Total genomic DNA was extracted from leaf samples, either fresh or dried with silica gel, using the DNeasy Plant Mini Kit (QIAGEN, Germany). Five cpDNA regions (*matK, rbcL-accD* IGS*, trnK-rps16* IGS*, ycf6-psbM* IGS and *psbA-trnH* IGS) and nDNA *pgiC* were amplified by polymerase chain reaction (PCR). Amplifications were carried out using a Veriti 96-Well Thermal Cycler (Applied Biosystems, USA) in 25 μL total volume containing 10–30 ng of genomic DNA, 1.25 U of *EF-Taq* DNA Polymerase (SolGent Co., Korea), 10 × *EF-Taq* Reaction Buffer with 25 mM MgCl_2_, 0.5 mM of each dNTP, 5% DMSO, and 0.4 μM of each primer. PCR and sequencing primers and PCR cycling conditions used in this study are provided in Table 3. The PCR products were purified using the enzymatic purification method^43^. Purified PCR products were sequenced using the BigDye Terminator v3.1 Cycle Sequencing Kit (Applied Biosystems, USA) following the manufacturer’s instructions. The sequence products were purified by ethanol precipitation and then run on an ABI 3730xl DNA Analyzer (Applied Biosystems, USA) at Macrogen Inc., Korea.

**Table 3:** PCR and sequencing primers and PCR cycling conditions for cpDNA *matK*, *rbcL-accD* IGS, *trnK–rps16* IGS, *ycf6-psbM* IGS and *psbA-trnH* IGS, and nDNA *pgiC*. Superscripts following primer names denote reference numbers. Primers pgiC_16F (5'-CAC AGC TTT TAC CAA CTG ATT C-3') and pgiC_21R (5'- CCT AAC TCA ACT CCC CAC-3') were designed by the authors.

Region	Primer	PCR cycling condition (35 cycles)				
		Predenaturation (3 min) (°C)	Denaturation (1 min) (°C)	Annealing (40 s) (°C)	Extension (1 min) (°C)	Final extension (7 min) (°C)
*pgiC*	pgiC16F	95	95	55	72	72
	pgiC21R					
*matK*	670F, 193F^44^	95	95	52	72	72
	1246R^44^					
*rbcL-accD* IGS	rbcL50F^45^	95	95	50	72	72
	accD79R^44^					
*trnK-rps16* IGS	trnKx1^46^	95	95	53	72	72
	rps16 × 2F2^46^					
*ycf6-psbM* IGS	ycf6F^47^	95	95	54	72	72
	psbMR^47^					
*psbA-trnH* IGS	psbAF^48^	95	95	55	72	72
	trnHR^48^					

### Cloning of nDNA pgiC

Due to the presence of polymorphic nucleotide positions in some direct sequences, PCR products of the nDNA *pgiC* region for 24 accessions of putative hybrids and *R. japonicus* were cloned using the pGEM-T Easy Vector System I (Promega, USA) following the manufacturer's instructions. Transformed *E. coli* cells were spread onto LB agar plates with ampicillin (50 µg/mL), and incubated at 37 °C for 12–16 h. Ten to 15 colonies per plate were randomly selected, and colonies were then screened for inserts by PCR. Temperature and cycling conditions consisted of lysis of cells and initial denaturation for 3 min at 95 °C, followed by 35 cycles of 1 min denaturation at 95 °C, 30 s annealing at 50 °C, and 1 min 15 s elongation at 72 °C, with a 7 min final extension at 72 °C. The PCR products were purified and sequenced using the same procedure described above.

### Sequence alignment and analyses

Nucleotide sequences were assembled and edited using Sequencher 5.4.6 (Gene Codes Corporation, USA). Edited sequences were aligned with Clustal X v. 2.0 with final manual adjustment using Se-Al v. 2.0a11^49,50^. All DNA sequences obtained in this study were deposited in GenBank (Supplementary Table S1). We identified a short inversion of 10 bp in the aligned sequence data set of *ycf6-psbM* IGS region. The inversion was replaced with the reverse complement of its sequence. As the inversion was assumed to be a single mutation event and appeared to have phylogenetic information, it was subsequently coded as a single binary character following the procedure suggested by Whitlock et al.^51^ In addition, six indels ranging from 1 to 9 bp in length in the cpDNA sequence data set and one 271-bp indel in the nDNA *pgiC* sequence data set were coded and added to the data matrices as extra binary characters^52^. Phylogenetic analyses were performed on the combined cpDNA sequence data sets using maximum parsimony (MP) and Bayesian inference (BI).

MP analyses were performed in PAUP*4.0a169^53^ using a heuristic search strategy with 100 random sequence additions, tree bisection-reconnection (TBR) branch swapping, ACCTRAN, MULTREES on, MAXTREE set to no limit, and HOLD = 10 in effect. All characters were treated as unordered and equally weighted, and gaps were treated as missing data except for seven indels and one inversion coded as binary characters. Bootstrap (BS) analyses of 1000 replicates were conducted in PAUP* to evaluate support for clades using the same search parameters as in the MP analyses above^54^. For BI analyses, the optimal model of sequence evolution for each data set was identified using the Akaike information criterion (AIC) in MrModeltest 2.3^55^. The following models of sequence evolution were identified as optimal for the five cpDNA regions examined in this study; GTR + I for *matK*, HKY for *rbcL-accD* IGS, GTR + G for *trnK-rps16* IGS and the combined cpDNA data set, GTR for *ycf6-psbM* IGS, F81 + I for *psbA-trnH* IGS. For nDNA *pgiC* region, the optimal model of sequence evolution was HKY + I. (Table 1). BI analyses were performed in MrBayes 3.2^56^ using two independent runs of four chains (three heated and one cold) for one million generations. Trees were sampled every 1000 generations, and the first 25% were discarded as burn-in. The remaining trees were used to produce a 50% majority-rule consensus tree and determine posterior probabilities (PP). An unrooted haplotype network was constructed for the cpDNA data set using the parsimony method as implemented in the TCS 1.21 program^57^. Gaps were treated as single evolutionary events and indels as the fifth state of character. The 95% probability limit of parsimonious connections was applied to produce the network.

## Supplementary Information

Supplementary Information.

## Data Availability

All sequence data have been deposited in GenBank.
