# Supplementary material for: Molecular evidence for natural hybridization between Rumex crispus and R. obtusifolius (Polygonaceae) in Korea
Source: Sci Rep. 2022 Mar 31;12:5423. doi: 10.1038/s41598-022-09292-9 (PMC8971494; doi:10.1038/s41598-022-09292-9)
Supplement: Supplementary file 1 — Supplementary Information. [file 41598_2022_9292_MOESM1_ESM.pdf]

Supplementary Information for:

Molecular evidence for natural hybridization between *Rumex crispus* and *R. obtusifolius* (Polygonaceae) in Korea

Gauri Shankar Bhandari, Chong-Wook Park*

School of Biological Sciences, Seoul National University, Seoul 08826, Korea.

SI Correspondence to:

Chong-Wook Park, parkc@snu.ac.kr

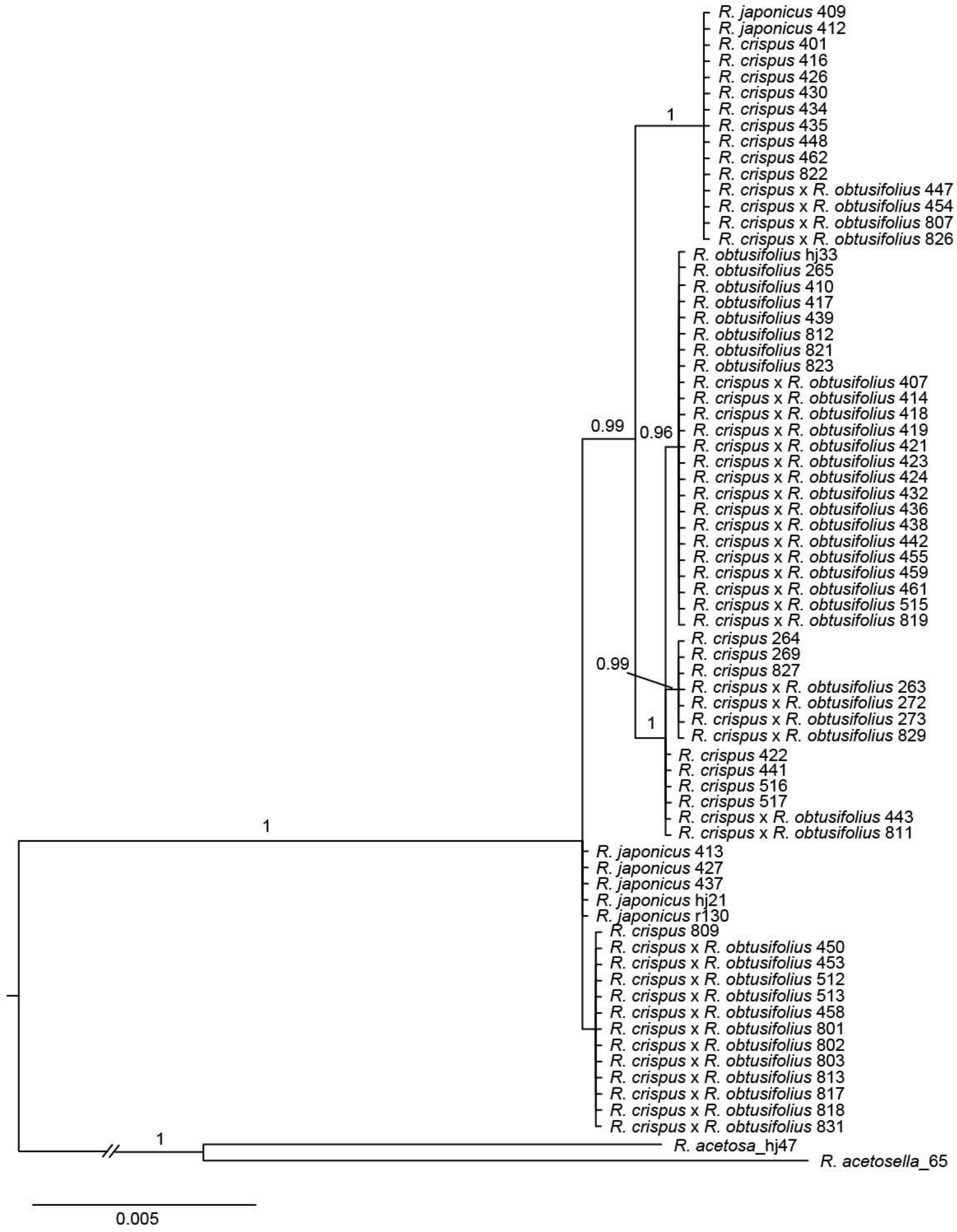

Figure S1. Bayesian majority-rule consensus tree of the combined cpDNA sequence data set for *Rumex crispus*, *R. obtusifolius*, *R. japonicus* and putative hybrids. Numbers above branches are Bayesian posterior probabilities (PP≥0.7). Accession numbers correspond to those in Supplementary Table S1.

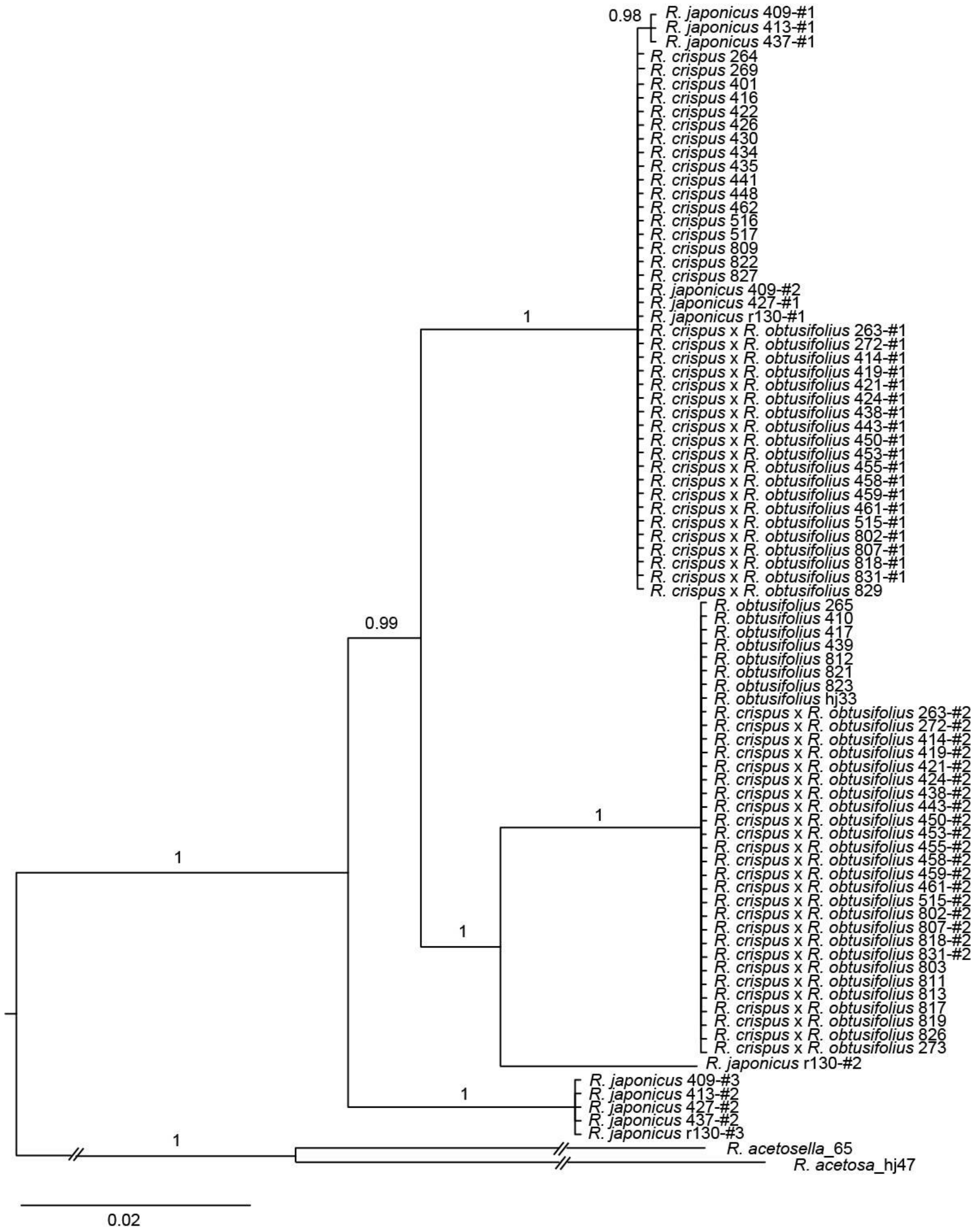

Figure S2. Bayesian majority-rule consensus tree of the nDNA *pgiC* sequence data set for *Rumex crispus*, *R. obtusifolius*, *R. japonicus* and putative hybrids. Numbers above branches are Bayesian posterior probabilities (PP≥0.7). Accession numbers correspond to those in Supplementary Table S1.

Table S1. Collection data and GenBank accession numbers for 87 accessions of *Rumex crispus*, *R. obtusifolius*, *R. japonicus* and putative hybrids examined in this study (outgroup taxa included).

Population	Locality	Taxon	Accession	cpDNA haplotype	GenBank accession number (*matK*, *rbcL-accD* IGS, *trnK-rps16* IGS, *ycf6-psbM* IGS, *psbA-trnH* IGS, *pgiC*). Missing *pgiC* sequences are indicated with a dash.
NS1	Naesosa, Jeon-buk, Korea	*R. crispus*	401	C1	OM927412, OM927646, OM927340, OM927484, OM927268, OM959623
		R. obtusifolius	410	C2	OM927440, OM927674, OM927368, OM927512, OM927296, OM927211
		R. japonicus	409	C1	OM927409, OM927643, OM927337, OM927481, OM927265, OM927183/OM927256/OM927261
		R. crispus x *R. obtusifolius*	407	C2	OM927443, OM927677, OM927371, OM927515, OM927299, –
		R. crispus x *R. obtusifolius*	801	C5	OM927429, OM927663, OM927357, OM927501, OM927285, –
		R. crispus x *R. obtusifolius*	802	C5	OM927425, OM927659, OM927353, OM927497, OM927281, OM927204/OM927248
		R. crispus x *R. obtusifolius*	803	C5	OM927436, OM927670, OM927364, OM927508, OM927292, OM927206
NS2	Naesosa, Jeon-buk, Korea	*R. crispus*	416	C1	OM927413, OM927647, OM927341, OM927485, OM927269, OM927219
		R. crispus	422	C4	OM927472, OM927706, OM927400, OM927544, OM927328, OM927220
		R. crispus	426	C1	OM927411, OM927645, OM927339, OM927483, OM927267, OM927221
		R. crispus	430	C1	OM927414, OM927648, OM927342, OM927486, OM927270, OM927222
		R. obtusifolius	417	C2	OM927453, OM927687, OM927381, OM927525, OM927309, OM927212
		R. japonicus	412	C1	OM927423, OM927657, OM927351, OM927495, OM927279, –
		R. japonicus	413	C6	OM927478, OM927712, OM927406, OM927550, OM927334, OM927181/OM927258
		R. japonicus	427	C6	OM927475, OM927709, OM927403, OM927547, OM927331, OM927236/OM927259
		R. crispus x *R. obtusifolius*	414	C2	OM927460, OM927694, OM927388, OM927532, OM927316, OM927185/OM927232
		R. crispus x *R. obtusifolius*	418	C2	OM927444, OM927678, OM927372, OM927516, OM927300, –
		R. crispus x *R. obtusifolius*	419	C2	OM927445, OM927679, OM927373, OM927517, OM927301, OM927186/OM927233

Table S1 (Continued).

Population	Locality	Taxon	Accession	cpDNA haplotype	GenBank accession number (*matK*, *rbcL-accD* IGS, *trnK-rps16* IGS, *ycf6-psbM* IGS, *psbA-trnH* IGS, *pgiC*). Missing *pgiC* sequences are indicated with a dash.
NS2	Naesosa, Jeon-buk, Korea	*R. crispus* x *R. obtusifolius*	421	C2	OM927457, OM927691, OM927385, OM927529, OM927313, OM927187/OM927234
		R. crispus x *R. obtusifolius*	423	C2	OM927442, OM927676, OM927370, OM927514, OM927298, –
		R. crispus x *R. obtusifolius*	424	C2	OM927446, OM927680, OM927374, OM927518, OM927302, OM927188/OM927235
		R. crispus x *R. obtusifolius*	432	C2	OM927447, OM927681, OM927375, OM927519, OM927303, –
NS3	Naesosa, Jeon-buk, Korea	*R. crispus*	434	C1	OM927415, OM927649, OM927343, OM927487, OM927271, OM927223
		R. crispus	435	C1	OM927416, OM927650, OM927344, OM927488, OM927272, OM927224
		R. japonicus	437	C6	OM927476, OM927710, OM927404, OM927548, OM927332, OM927182/OM927260
		R. obtusifolius	439	C2	OM927439, OM927673, OM927367, OM927511, OM927295, OM927213
		R. crispus x *R. obtusifolius*	436	C2	OM927458, OM927692, OM927386, OM927530, OM927314, –
		R. crispus x *R. obtusifolius*	438	C2	OM927459, OM927693, OM927387, OM927531, OM927315, OM927189/OM927237
NS4	Naesosa, Jeon-buk, Korea	*R. crispus*	809	C5	OM927424, OM927658, OM927352, OM927496, OM927280, OM927231
		R. crispus	822	C1	OM927422, OM927656, OM927350, OM927494, OM927278, OM927249
		R. crispus	516	C4	OM927469, OM927703, OM927397, OM927541, OM927325, OM927250
		R. crispus	517	C4	OM927471, OM927705, OM927399, OM927543, OM927327, OM927251
		R. crispus	441	C4	OM927473, OM927707, OM927401, OM927545, OM927329, OM927225
		R. obtusifolius	823	C2	OM927455, OM927689, OM927383, OM927527, OM927311, OM927216
		R. obtusifolius	hj33	C2	OM927438, OM927672, OM927366, OM927510, OM927294, OM927217
		R. japonicus	hj21	C6	OM927477, OM927711, OM927405, OM927549, OM927333, –
		R. japonicus	r130	C6	OM927474, OM927708, OM927402, OM927546, OM927330, OM927218/OM927257/OM927262
		R. crispus x *R. obtusifolius*	442	C2	OM927448, OM927682, OM927376, OM927520, OM927304, –

Table S1 (Continued).

Population	Locality	Taxon	Accession	cpDNA haplotype	GenBank accession number (*matK*, *rbcL-accD* IGS, *trnK-rps16* IGS, *ycf6-psbM* IGS, *psbA-trnH* IGS, *pgiC*). Missing *pgiC* sequences are indicated with a dash.
NS4	Naesosa, Jeon-buk, Korea	*R. crispus* x *R. obtusifolius*	442	C2	OM927448, OM927682, OM927376, OM927520, OM927304, –
		R. crispus x *R. obtusifolius*	443	C4	OM927470, OM927704, OM927398, OM927542, OM927326, OM927190/OM927238
		R. crispus x *R. obtusifolius*	512	C5	OM927426, OM927660, OM927354, OM927498, OM927282, –
		R. crispus x *R. obtusifolius*	515	C2	OM927452, OM927686, OM927380, OM927524, OM927308, OM927209/OM927255
		R. crispus x *R. obtusifolius*	513	C5	OM927427, OM927661, OM927355, OM927499, OM927283, –
		R. crispus x *R. obtusifolius*	807	C1	OM927421, OM927655, OM927349, OM927493, OM927277, OM927208/OM927254
		R. crispus x *R. obtusifolius*	811	C4	OM927468, OM927702, OM927396, OM927540, OM927324, OM927199
		R. crispus x *R. obtusifolius*	813	C5	OM927435, OM927669, OM927363, OM927507, OM927291, OM927200
		R. crispus x *R. obtusifolius*	817	C5	OM927434, OM927668, OM927362, OM927506, OM927290, OM927201
		R. crispus x *R. obtusifolius*	818	C5	OM927428, OM927662, OM927356, OM927500, OM927284, OM927202/OM927247
		R. crispus	827	C3	OM927461, OM927695, OM927389, OM927533, OM927317, OM927230
		R. crispus x *R. obtusifolius*	829	C3	OM927462, OM927696, OM927390, OM927534, OM927318, OM927252
		R. crispus x *R. obtusifolius*	819	C2	OM927454, OM927688, OM927382, OM927526, OM927310, OM927203
NS5	Naesosa, Jeon-buk, Korea	*R. crispus*	448	C1	OM927418, OM927652, OM927346, OM927490, OM927274, OM927226
		R. obtusifolius	821	C2	OM927437, OM927671, OM927365, OM927509, OM927293, OM927215
		R. crispus x *R. obtusifolius*	450	C5	OM927433, OM927667, OM927361, OM927505, OM927289, OM927191/OM927239
		R. crispus x *R. obtusifolius*	453	C5	OM927432, OM927666, OM927360, OM927504, OM927288, OM927192/OM927240
		R. crispus x *R. obtusifolius*	447	C1	OM927417, OM927651, OM927345, OM927489, OM927273, –
		R. crispus x *R. obtusifolius*	454	C1	OM927419, OM927653, OM927347, OM927491, OM927275, –

Table S1 (Continued).

Population	Locality	Taxon	Accession	cpDNA haplotype	GenBank accession number (*matK*, *rbcL-accD* IGS, *trnK-rps16* IGS, *ycf6-psbM* IGS, *psbA-trnH* IGS, *pgiC*). Missing *pgiC* sequences are indicated with a dash.
NS5	Naesosa, Jeon-buk, Korea	*R. crispus* x *R. obtusifolius*	455	C2	OM927449, OM927683, OM927377, OM927521, OM927305, OM927193/OM927241
		R. crispus x *R. obtusifolius*	826	C1	OM927410, OM927644, OM927338, OM927482, OM927266, OM927205
NS6	Naesosa, Jeon-buk, Korea	*R. crispus*	462	C1	OM927420, OM927654, OM927348, OM927492, OM927276, OM927227
		R. obtusifolius	812	C2	OM927456, OM927690, OM927384, OM927528, OM927312, OM927214
		R. crispus x *R. obtusifolius*	458	C5	OM927430, OM927664, OM927358, OM927502, OM927286, OM927194/OM927242
		R. crispus x *R. obtusifolius*	459	C2	OM927450, OM927684, OM927378, OM927522, OM927306, OM927195/OM927243
		R. crispus x *R. obtusifolius*	461	C2	OM927451, OM927685, OM927379, OM927523, OM927307, OM927196/OM927244
		R. crispus x *R. obtusifolius*	831	C5	OM927431, OM927665, OM927359, OM927503, OM927287, OM927207/OM927253
GD1	Gadeok-do, Busan, Korea	*R. crispus*	264	C3	OM927464, OM927698, OM927392, OM927536, OM927320, OM927228
		R. crispus	269	C3	OM927465, OM927699, OM927393, OM927537, OM927321, OM927229
		R. obtusifolius	265	C2	OM927441, OM927675, OM927369, OM927513, OM927297, OM927210
		R. crispus x *R. obtusifolius*	263	C3	OM927463, OM927697, OM927391, OM927535, OM927319, OM927197/OM927245
		R. crispus x *R. obtusifolius*	272	C3	OM927466, OM927700, OM927394, OM927538, OM927322, OM927198/OM927246
		R. crispus x *R. obtusifolius*	273	C3	OM927467, OM927701, OM927395, OM927539, OM927323, OM927184
SW1	Suwon, Korea	*R. crispus*	318	C5	OM927554, OM927569, OM927584, OM927599, OM927614, OM927635
		R. crispus	319	C5	OM927553, OM927568, OM927583, OM927598, OM927613, OM927634
		R. crispus	324	C3	OM927565, OM927580, OM927595, OM927610, OM927625, OM927640
SW2	Suwon, Korea	*R. obtusifolius*	006	C2	OM927560, OM927575, OM927590, OM927605, OM927620, OM927630

Table S1 (Continued).

Population	Locality	Taxon	Accession	cpDNA haplotype	GenBank accession number (*matK*, *rbcL-accD* IGS, *trnK-rps16* IGS, *ycf6-psbM* IGS, *psbA-trnH* IGS, *pgiC*). Missing *pgiC* sequences are indicated with a dash.
SH1	Siheung, Gyeonggi, Korea	*R. crispus*	910	C5	OM927556, OM927571, OM927586, OM927601, OM927616, OM927637
		R. crispus	911	C5	OM927555, OM927571, OM927586, OM927601, OM927616, OM927637
		R. crispus	934	C5	OM927557, OM927572, OM927587, OM927602, OM927617, OM927638
SE1	Seoul, Korea	*R. obtusifolius*	007	C2	OM927561, OM927576, OM927591, OM927606, OM927621, OM927631
NY1	Namyang-ju, Gyeonggi, Korea	*R. crispus*	207	C3	OM927564, OM927579, OM927594, OM927609, OM927624, OM927639
WD1	Wan-do, Jeon-nam, Korea	*R. crispus*	122	C5	OM927567, OM927582, OM927597, OM927612, OM927627, OM927642
NG1	Gyeong-nam, Korea	*R. crispus*	hj26	C4	OM927566, OM927581, OM927596, OM927611, OM927626, OM927641
JS1	Jeon-nam, Korea	*R. obtusifolius*	001	C2	OM927558, OM927573, OM927588, OM927603, OM927618, OM927628
NR1	Ulleung-do, Gyeong-buk Korea	*R. obtusifolius*	002	C2	OM927559, OM927574, OM927589, OM927604, OM927619, OM927629
NR2	Ulleung-do, Gyeong-buk, Korea	*R. obtusifolius*	011	C2	OM927562, OM927577, OM927592, OM927607, OM927622, OM927632
		R. obtusifolius	012	C2	OM927563, OM927578, OM927593, OM927608, OM927623, OM927633
Outgroup	Wando, Jeon-nam, Korea	*R. acetosa*	hj47		OM927480, OM927714, OM927408, OM927552, OM927336, OM927263
Outgroup	Seoul, Korea	*R. acetosella*	65		OM927479, OM927713, OM927407, OM927551, OM927335, OM927264

Table S2. Variable nucleotide sites for five nDNA *pgiC* haplotypes recovered from 85 accessions of *Rumex crispus*, *R. obtusifolius*, *R. japonicus* and putative hybrids examined in this study.

	Variable nucleotide sites in alignment																																														
nDNA *pgiC* haplotype	0017	0026	0031	0041	0052	0053	0067	0074	0082	0090	0091	0092	0093	0094	0095	0096	0097	0098	0099	0103	0110	0111	0112	0119	0120	0126	0128	0133	0138	0139	0140	0141	0142	0143	0144	0145	0146	0147	0158	0163	0167	0169	0171	0183	0184	0188	0194	0253
N1	C	G	G	G	T	A	A	A	T	A	A	C	T	A	A	-	-	-	-	G	T	T	A	G	C	T	G	A	C	A	T	G	G	C	T	C	C	T	T	A	C	G	T	G	T	A	G	C
N2	C	G	G	G	T	A	A	A	T	A	A	C	T	A	A	-	-	-	-	G	T	T	A	G	C	T	G	A	C	A	T	G	G	C	T	C	C	T	T	A	C	G	T	G	T	A	G	C
N3	C	G	T	T	-	-	T	A	C	A	-	-	-	-	-	-	-	-	-	C	T	T	A	C	T	T	G	C	C	A	T	G	G	C	T	C	C	T	T	A	T	G	T	G	C	G	G	G
N4	C	G	T	G	-	-	G	A	C	A	A	C	C	A	A	-	-	-	-	G	T	T	A	G	T	T	T	A	-	-	-	-	-	-	-	-	-	-	T	T	T	A	C	A	T	A	G	C
N5	T	C	G	G	-	-	G	C	C	G	A	C	C	A	A	T	A	A	A	G	-	-	-	G	C	C	T	A	C	A	T	G	G	C	T	C	C	T	A	A	T	G	T	G	T	A	C	C

	Variable nucleotide sites in alignment																																														
nDNA *pgiC* haplotype	0262	0270	0284	0287	0288	0301	0303	0304	0306	0320	0321	0322	0323	0324	0325	0356	0330	0337	0345	0366	-	0633	0637	0659	0671	0680	0713	0722	0733	0734	0735	0740	0746	0750	0752	0753	0764	0765	0766	0767	0768	0769	0770	0771	0772	0773	0774	0775
N1	G	A	C	C	A	G	A	T	T	-	-	-	-	-	-	-	A	C	T		(271 bp)		A	C	T	T	G	T	A	C	A	C	A	-	C	T	T	A	A	C	T	T	C	T	T	T	T	A
N2	G	A	C	C	A	G	A	T	T	-	-	-	-	-	-	-	A	C	T		-		A	C	T	T	G	T	A	C	A	C	A	-	C	T	T	A	A	C	T	T	C	T	T	T	T	A
N3	C	C	G	C	T	G	-	C	T	G	G	C	A	G	T	A	A	T	C		-		T	C	A	T	G	C	A	C	G	T	T	T	C	C	T	A	A	C	T	T	C	T	T	T	T	A
N4	C	C	G	T	A	G	A	T	G	G	G	C	A	G	T	A	T	C	C		-		T	T	A	T	A	C	G	A	A	T	A	-	C	C	T	A	A	C	T	T	C	T	T	T	T	A
N5	C	A	G	C	T	A	A	T	T	G	G	C	G	G	T	A	A	C	T		-		T	C	A	C	G	T	A	C	A	T	A	-	T	C	-	-	-	-	-	-	-	-	-	-	-	-

Table S2 (Continued).

nDNA *pgiC* haplotype	Variable nucleotide sites in alignment																																														
	0776	0777	0778	0779	0780	0781	0782	0783	0784	0785	0786	0801	0807	0848	0859	0867	0887	0888	0892	0896	0897	0898	0899	0922	0925	0926	0930	0931	0932	0933	0934	0937	0939	0940	0947	0954	0956	0959	0963	1011	1020	1039	1062	1079	1085	1086	1087	1088
N1	T	G	A	A	C	T	A	G	A	A	A	A	C	T	A	T	T	C	A	T	T	T	A	G	G	G	-	-	-	-	-	A	T	T	G	T	A	A	C	G	T	A	A	T	-	-	-	-
N2	T	G	A	A	C	T	A	G	A	A	A	A	C	T	A	T	T	C	A	T	T	T	A	G	G	G	-	-	-	-	-	A	T	T	G	T	A	A	C	G	T	A	A	T	-	-	-	-
N3	T	A	A	A	C	T	A	G	A	A	A	T	C	T	A	T	T	C	G	C	T	T	-	G	A	C	C	T	T	-	-	T	A	A	A	T	G	A	C	C	T	G	-	T	A	C	A	C
N4	T	A	A	A	T	T	A	G	A	A	A	A	T	T	A	T	T	C	G	C	T	T	A	G	A	C	C	-	T	-	-	T	T	T	G	T	G	C	C	C	T	A	-	G	A	C	A	C
N5	-	-	-	-	-	-	-	-	-	-	-	A	C	C	C	C	C	T	G	-	-	-	-	T	G	C	-	-	-	T	T	T	T	T	G	C	G	A	T	C	C	A	A	T	A	C	A	C

nDNA *pgiC* haplotype	Variable nucleotide sites in alignment																																														
	1089	1090	1091	1092	1093	1094	1095	1096	1097	1098	1099	1100	1103	1106	1126	1129	1130	1131	1141	1142	1143	1144	1145	1153	1154	1160	1176	1177	1183	1190	1191	1268	1274	1281	1282	1294	1317	1322	1328	1336	1340	1341	1342	1346	1351	1352	1358	1381
N1	-	-	-	-	-	-	-	-	-	-	-	-	T	T	G	C	T	A	T	T	T	C	T	C	A	G	A	A	A	-	-	G	G	G	G	A	C	A	C	G	C	A	C	A	G	C	T	T
N2	-	-	-	-	-	-	-	-	-	-	-	-	T	T	G	C	T	A	T	T	T	C	T	C	A	G	A	A	A	-	-	G	G	G	G	A	C	A	C	G	C	A	C	A	G	C	T	T
N3	T	C	C	A	T	T	A	A	A	G	T	T	T	T	G	A	G	-	T	T	T	C	C	T	C	G	A	A	G	C	C	G	T	A	A	A	T	G	T	G	T	C	C	A	A	C	T	T
N4	T	C	C	A	T	T	A	A	A	G	T	T	T	A	A	A	G	-	T	T	T	C	C	T	C	G	A	T	G	C	C	G	G	G	G	G	T	G	C	G	T	C	T	T	G	T	T	T
N5	T	C	C	A	T	T	A	T	A	G	T	T	A	T	G	A	A	A	-	-	-	-	-	T	C	C	G	T	G	-	-	T	G	G	G	A	T	A	C	C	C	C	T	A	G	C	C	G
